# A 6-Year Update on the Diversity of Methicillin-Resistant *Staphylococcus aureus* Clones in Africa: A Systematic Review

**DOI:** 10.3389/fmicb.2022.860436

**Published:** 2022-05-03

**Authors:** Opeyemi Uwangbaoje Lawal, Olaniyi Ayobami, Alaa Abouelfetouh, Nadira Mourabit, Mamadou Kaba, Beverly Egyir, Shima M. Abdulgader, Adebayo Osagie Shittu

**Affiliations:** ^1^Laboratory of Bacterial Evolution and Molecular Epidemiology, Instituto de Tecnologia Química e Biológica, Universidade Nova de Lisboa (ITQB-NOVA), Oeiras, Portugal; ^2^Unit for Healthcare-Associated Infections, Surveillance of Antimicrobial Resistance and Consumption, Robert Koch Institute, Berlin, Germany; ^3^Department of Microbiology and Immunology, Faculty of Pharmacy, Alexandria University, Alexandria, Egypt; ^4^Department of Microbiology and Immunology, Faculty of Pharmacy, AlAlamein International University, Alalamein, Egypt; ^5^Biotechnology, Environmental Technology and Valorisation of Bio-Resources Team, Department of Biology, Faculty of Sciences and Techniques of Al Hoceima, Abdelmalek Essaadi University, Tetouan, Morocco; ^6^Division of Medical Microbiology, Department of Clinical Laboratory Sciences, Faculty of Health Sciences, University of Cape Town, Cape Town, South Africa; ^7^Department of Bacteriology, Noguchi Memorial Institute for Medical Research, College of Health Sciences, University of Ghana, Accra, Ghana; ^8^Division of Molecular Biology and Human Genetics, Faculty of Medicine and Health Sciences, DSI-NRF Centre of Excellence for Biomedical Tuberculosis Research, South African Medical Research Council Centre for Tuberculosis Research, Stellenbosch University, Cape Town, South Africa; ^9^Department of Microbiology, Obafemi Awolowo University, Ile-Ife, Nigeria; ^10^Institute of Medical Microbiology, University Hospital Münster, Münster, Germany

**Keywords:** MRSA – methicillin-resistant *Staphylococcus aureus*, clonal complex (CC), Panton–Valentine leukocidin (PVL), molecular typing, Africa

## Abstract

**Background:**

Methicillin-resistant *Staphylococcus aureus* (MRSA) is a leading cause of hospital-associated (HA) and community-associated (CA) infections globally. The multi-drug resistant nature of this pathogen and its capacity to cause outbreaks in hospital and community settings highlight the need for effective interventions, including its surveillance for prevention and control. This study provides an update on the clonal distribution of MRSA in Africa.

**Methods:**

A systematic review was conducted by screening for eligible English, French, and Arabic articles from November 2014 to December 2020, using six electronic databases (PubMed, EBSCOhost, Web of Science, Scopus, African Journals Online, and Google Scholar). Data were retrieved and analyzed according to the Preferred Reporting Items for Systematic Review and Meta-Analysis guidelines (registered at PROSPERO: CRD42021277238). Genotyping data was based primarily on multilocus sequence types (STs) and Staphylococcal Cassette Chromosome *mec* (SCC*mec*) types. We utilized the Phyloviz algorithm in the cluster analysis and categorization of the MRSA STs into various clonal complexes (CCs).

**Results:**

We identified 65 studies and 26 publications from 16 of 54 (30%) African countries that provided sufficient genotyping data. MRSA with diverse staphylococcal protein A (*spa*) and SCC*mec* types in CC5 and CC8 were reported across the continent. The ST5-IV [2B] and ST8-IV [2B] were dominant clones in Angola and the Democratic Republic of Congo (DRC), respectively. Also, ST88-IV [2B] was widely distributed across the continent, particularly in three Portuguese-speaking countries (Angola, Cape Verde, and São Tomé and Príncipe). The ST80-IV [2B] was described in Algeria and Egypt, while the HA-ST239/ST241-III [3A] was only identified in Egypt, Ghana, Kenya, and South Africa. ST152-MRSA was documented in the DRC, Kenya, Nigeria, and South Africa. Panton–Valentine leukocidin (PVL)-positive MRSA was observed in several CCs across the continent. The median prevalence of PVL-positive MRSA was 33% (ranged from 0 to 77%; *n* = 15).

**Conclusion:**

We observed an increase in the distribution of ST1, ST22, and ST152, but a decline of ST239/241 in Africa. Data on MRSA clones in Africa is still limited. There is a need to strengthen genomic surveillance capacity based on a “One-Health” strategy to prevent and control MRSA in Africa.

## Background

Methicillin-resistant *Staphylococcus aureus* (MRSA) is one of the important antibiotic-resistant pathogens and a leading cause of hospital-associated (HA) and community-associated (CA) infections worldwide (Lee et al., 2018). Recently, the World Health Organization (WHO) included MRSA as one of the indicators for antimicrobial resistance in the Sustainable Development Goals connected to the health target 3.d (WHO, 2021). MRSA is a major burden in hospital-acquired neonatal infections in sub-Saharan Africa (Okomo et al., 2019). Vancomycin, a glycopeptide, is considered one of the last therapeutic agents for MRSA infections (McGuinness et al., 2017). However, MRSA isolates from clinical samples exhibiting reduced susceptibility to vancomycin have been documented in Africa (Fortuin-de Smidt et al., 2015; Zorgani et al., 2015; Eshetie et al., 2016; Bamigboye et al., 2018; ElSayed et al., 2018). In addition, *mecA*-positive (Lozano et al., 2016), *mecC*-positive MRSA (Dweba and Zishiri, 2019), and vancomycin-resistant (*vanA*, *vanB*-positive) MRSA (Al-Amery et al., 2019) have been identified in food animals on the African continent.

There are varying prevalence rates of MRSA reported in Africa (Wangai et al., 2019), and the epidemiological picture depicts diverse clonal types within regions and countries. We published a systematic review on the molecular epidemiology of MRSA in Africa (Abdulgader et al., 2015). It revealed that the pandemic MRSA clones: sequence type (ST) 5 and ST239/241 were dominant on the continent. However, some clones were limited to specific countries (e.g., ST612 in South Africa) or regions (ST80 in North Africa). Moreover, CA-MRSA (ST8 and ST88) were identified in clinical and non-clinical settings (Abdulgader et al., 2015). Africa is described as a Panton–Valentine leukocidin (PVL) endemic region (Schaumburg et al., 2014). Also, the 2015 review observed a PVL prevalence of 0.3–100% among MRSA identified from humans (carriage and infection) in Africa. Despite these findings, data is still limited, and there are knowledge gaps on the clonal nature of MRSA in Africa.

The epidemiology of MRSA is characterized by the occurrence and dissemination of new and emerging clones leading to constant changes globally (Turner et al., 2019). For instance, a steady increase of ST5 and ST93 as the predominant CA-MRSA clones have been described in Australia (Bloomfield et al., 2020), and ST59 has been replaced by ST239 in China (Li et al., 2018). Furthermore, a decline of ST5 and an increase in ST8 cases have been observed in the United States of America (See et al., 2020) and Canada (Guthrie et al., 2020). Since MRSA is a significant public health problem, understanding the changes in epidemiology through regular monitoring and surveillance is essential to minimize its healthcare and economic burden. Therefore, this review aimed to provide an update describing the clonal characteristics of MRSA in Africa.

## Methods

This systematic review is a 6-year update on the MRSA clonal diversity in Africa. We performed the systematic literature search and analysis according to the Preferred Reporting Items for Systematic Reviews and Meta-Analyses (PRISMA) (Page et al., 2021). The study was registered in the PROSPERO database (CRD42021277238). Since this review focused on a narrative description of the eligible studies instead of effect sizes and other related quantitative outcomes, methodological features like sample size, study population, use of appropriate study design were not assessed. Therefore, we did not do a formal risk of bias scoring system.

### Literature Search Approach

We used six electronic databases to identify and retrieve relevant information (PubMed, EBSCOhost, Web of Science, Scopus, African Journals Online, and Google Scholar). The search included articles published in English, French, and Arabic from November 01, 2014, to December 31, 2020. The literature search date was selected to complement the data previously described (Abdulgader et al., 2015). The literature search was also complemented with Publish or perish literature and citation mining algorithm (Harzing, 2007).

Predefined search terms were used (Supplementary Table 1), first on a continent-wide basis and then for the 54 African countries. Article titles and abstracts were screened and reviewed independently by two authors (OL, AS), including full-text reviews on all eligible studies.

### Identification of Eligible Studies

Studies were eligible on the condition that identification of MRSA was based primarily on the molecular detection of the methicillin resistance (*mecA*) gene (including *mecC*), and the investigations used at least one molecular tool to characterize the isolates. We also included global surveys that involved African countries. All duplicate articles were removed, and data only on phenotypic antibiotic susceptibility testing to identify methicillin-susceptible *Staphylococcus aureus* (MSSA) and *mecA* were excluded. Moreover, African studies that described isolates recovered from humans or animals not resident on the continent were excluded. Sufficient genotyping data was based primarily on multilocus sequence type (MLST) and the Staphylococcal Cassette Chromosome *mec* (SCC*mec*) typing nomenclature as previously reported (Abdulgader et al., 2015). Also, we included additional data, e.g., staphylococcal protein A (*spa*) types and PVL status. The MRSA STs cluster analysis was performed and categorized into various clonal complexes (CCs) using Phyloviz version 2.0.^1^

### Data Extraction and Analyses

We extracted the epidemiological and genotypic data of MRSA from the eligible articles using standardized forms. Publications that described a previously analyzed collection within the period under review were considered as a single study. We determined the PVL rate from eligible studies with a sample size of ≥30 MRSA isolates.

### Cluster Analysis and Minimum Spanning Tree

The relationship between the MRSA STs described in this review with other common lineages reported worldwide was analyzed as previously described (Abdulgader et al., 2015). Briefly, we downloaded the allelic profiles of the African MRSA STs from the MLST website.^2^ Furthermore, 236 randomly selected STs representing the diversity in the database and based on the differences in their allelic profiles were included (Supplementary Table 2). The minimum spanning tree was constructed with the goeBURST algorithm using the Phyloviz version 2.0 (see text footnote 1).

## Results

### Literature Search and Description of the Articles Included in the Review

**FIGURE 1:**
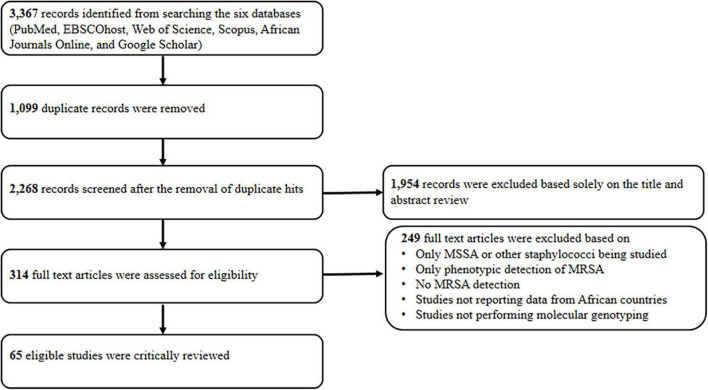
Standard preferred reporting item for systematic reviews. MSSA, methicillin susceptible *Staphylococcus aureus*; MRSA, methicillin-resistant *Staphylococcus aureus*.

The systematic search yielded 3367 articles (Figure 1). We screened 314 full-text articles after removing duplicate studies and assessing titles and abstracts. Overall, 65 studies were considered eligible for the qualitative analysis. The data from these studies were obtained from investigations conducted in 22 countries. Most of the single-center studies were from Egypt (*n* = 9), Nigeria (*n* = 9), South Africa (*n* = 8), Algeria (*n* = 6), and Ghana (*n* = 5) (Table 1). Multicentre studies were from six reports. They included four investigations in Portuguese-speaking African countries: Angola, Cape Verde, and São Tomé and Príncipe (Conceição et al., 2015a,b; Aires-de-Sousa et al., 2018; Rodrigues et al., 2018). Others were one study each from Cameroon and South Africa (Founou et al., 2019), Cote d’Ivoire and the Democratic Republic of Congo (DRC) (Schaumburg et al., 2015).

**TABLE 1:** Summary of the characteristics of eligible articles on the molecular epidemiology of methicillin-resistant *Staphylococcus aureus* (MRSA) in Africa.

Country	Study period	Sample type	Host	No of *S. aureus* isolates	*Staphylococcus aureus* ID	No of MRSA	Settings	Molecular typing methods							Detection of genes			References
								*coa*	*agr*	*spa* typing	PFGE	SCC*mec*	MLST	WGS	PVL	*Virulence	Antibiotic resistance	
Algeria	2010–2012	Nasal swabs	Human	159	NR	9	HA	–	–	–	–	✓	✓	–	✓	✓	–	Djoudi et al., 2014
	2011–2012	NR	Human	NR	NR	99	NR	–	✓	✓	–	–	✓	–	–	✓	✓	Elhani et al., 2015
	2015–2016	Nasal swabs from animals	Camel, horses, sheep, monkeys, cattle	118	MALDI-TOF	6	LA	–	✓	–	–	✓	✓	–	✓	✓	✓	Agabou et al., 2017
	2014–2015	Diverse raw and processed food products	Food	153	MALDI-TOF	26	CA	–	–	–	–	✓	–	–	✓	✓	–	Chaalal et al., 2018
	2014–2015	Raw milk	Cows	69	23 rRNA gene PCR	11	CA	–	–	✓	✓	–	✓	–	✓	✓	✓	Titouche et al., 2019
	2017–2018	Dairy and meat samples	Animal	104	23 rRNA	5	CA	–	–	✓	–	–	✓	–	✓	✓	✓	Titouche et al., 2020
DR Congo	2013–2014	SSTI, UTI, ear-eye-nose-throat infection, blood	Human	186	NR	55	HA	–	✓	✓	–	✓	✓	✓	✓	✓	✓	Lebughe et al., 2017¶
	2009–2012	Blood samples	Human	108	NR	27	HA	–	–	✓	–	✓	✓	–	✓	✓	✓	Vandendriessche et al., 2017
Egypt	2010–2012	Human: pus, sputum, urine, cerebrospinal fluid, swabs, mastitic cow milk	Human, mastitic cow	133	*nuc* gene PCR	30	HA/LA	✓	–	✓	–	✓	–	–	✓	–	–	Abd El-Hamid and Bendary, 2015
	2011	Nasal swabs	Human	54	*nuc* gene PCR	33	CA	–	–	✓	–	✓	–	–	✓	–	–	Abou Shady et al., 2015
	2014	Diabetic foot, nasal discharge, boils, abscesses, sputum, urine, wounds, burns, vaginal smear	Human	136	NR	85	HA	✓	✓	–	–	✓	–	–	–	✓	–	El-baz et al., 2017
	2013	Nasal swabs of health care workers, hospital environmental surfaces	Human	112	16S rRNA gene PCR	34	HA	–	–	✓	–	✓	–	–	✓	✓	✓	Khairalla et al., 2017
	2016–2017	Human: pus, blood, cerebrospinal fluid, pericardial fluid, sputum, urine, swabs from human; Sheep and cow: pus, meat, and milk from mastitic animals	Human, sheep, and cows	65	16S rRNA and *nuc* gene PCR	65	HA [20] LA [22], CA [23]	✓	✓	✓	–	✓	✓	–	✓	✓	✓	Abd El-Hamid et al., 2019
	NR	Clinical and milk samples from mastitic cow	Cows	17	*nuc* gene PCR	5	LA	–	–	–	–	–	✓	–	–	–	–	Oreiby et al., 2019
	2014–2016	Blood, sputum, and pus	Human	NR	*nuc* gene PCR	120	HA [80], CA [40]	–	–	–	–	✓	–	–	✓	–	–	Shehata et al., 2019
	2017–2018	Milk from mastitic cow	Cows	42	MALDI-TOF	12	LA	–	✓	✓	–	–	–	–	✓	✓	✓	El-Ashker et al., 2020
	2017–2018	Diverse samples from ICU	Human	NR	NR	18	HA	–	–	–	–	✓	✓	✓	✓	✓	✓	Soliman et al., 2020¶
Ethiopia	2016–2017	Nasal swabs from workers and cow udder	Farm workers/ cows	70	*nuc* gene PCR	1	LA	–	–	✓	–	✓	–	–	–	–	–	Kalayu et al., 2020
	2014–2018	Blood, wound lesions	Human	80	MALDI-TOF, 16S rRNA PCR	1	HA	–	–	–	–	✓	✓	–	✓	✓	–	Verdú-Expósito et al., 2020
Gabon	2012–2013	Throat swabs, skin lesions	Human	103	NR	3	CA	–	–	–	–	✓	–	–	✓	✓	✓	Okuda et al., 2016
Ghana	2010–2013	Clinical samples, nasal swabs	Human	24	Microarray	24	HA, CA	✓	✓	✓	–	✓	✓	–	✓	–	–	Egyir et al., 2015
	2007–2012	Blood, sputum, and pus	Human	56	MALDI-TOF, *nuc* gene PCR	1	HA	–	–	✓	–	–	–	–	✓	–	–	Dekker et al., 2016
	2014–2015	Nasal swabs	Human	123	NR	2	HA	–	–	✓	–	–	✓	–	✓	✓	–	Eibach et al., 2017
	NR	Nasal swabs from cattle, pigs, goats, sheep, and handlers	Human/ animals	25	MALDI-TOF	2	CA	–	–	✓	–	–	✓	✓	✓	✓	✓	Egyir et al., 2020
	2016	Wound	Human	28	NR	8	HA	–	–	–	–	✓	✓	✓	✓	–	✓	Wolters et al., 2020¶
Kenya	NR	Nasal swabs, pus, blood, tracheal aspirate, axillary swab, ear swab, sputum, vulva swabs	Human	93	NR	32	HA	–	–	✓	✓	✓	✓	–	–	–	–	Omuse et al., 2016
	2015–2018	NR	Human	32	VITEK 2	8	HA	–	–	✓	–	✓	✓	✓	–	✓	✓	Kyany’a et al., 2019¶
Libya	2008, 2014	Swabs; nose, ears, wounds, throat; pus, sputum, urine	Human	NR	NR	95	HA/CA	–	–	✓	–	–	✓	–	✓	–	–	Ahmed et al., 2017
	2013	Wound	Human	NR	*nuc* gene PCR	32	HA	–	–	–	✓	✓	✓	–	✓	✓	✓	Khemiri et al., 2017
Madagascar	NR	Nasal swabs	Human	171	*nuc* gene PCR	20	HA [14], CA [6]	–	–	✓	–	–	–	–	✓	✓	–	Hogan et al., 2016
Morocco	2012–2013	Nasal swabs	Human	400	16S rRNA and *nuc* gene PCR	17	CA	–	–	✓	✓	✓	✓	–	✓	✓	–	Mourabit et al., 2017
Nigeria	2013	Clinical samples	Human	156	API 20	66	HA	–	–	–	–	✓	–	–	✓	✓	–	Alli et al., 2015
	2010–2011	Nasal swabs, wounds, vaginal discharge, blood, urine, sputum	Human	290	*nuc* gene PCR	7	HA [5], CA [2]	–	–	✓	–	–	–	–	✓	–	–	Ayepola et al., 2015
	NR	Cloacal samples from birds	Birds	247	Staph Latex Agglutination	15 (subsampled 8 MRSA isolates)	LA	–	–	✓	–	✓	✓	–	✓	–	✓	Nworie et al., 2017
	NR	Blood, urine, wound, sputum	Human	92	VITEK 2	12	HA	–	–	–	–	✓	–	–	✓	–	–	Enwuru et al., 2018
	NR	Nasal swabs from food animals and abattoir workers and environmental samples	Human and animals	109	MALDI-TOF and *tuf* gene PCR	18	LA	–	–	✓	–	–	–	–	–	–	–	Odetokun et al., 2018
	2013–2015	Nasal swabs	Pigs/human	NR	MALDI-TOF	38	LA [26], CA [12]	–	–	✓	–	✓	✓	✓	✓	✓	–	Otalu et al., 2018¶
	NR	Diverse samples from humans, animals, and animal products	Human, animals, and chicken in a poultry farm	61	MALDI-TOF	56 (subsampled 30 MRSA isolates)	LA	–	–	✓	–	✓	✓	✓	✓	✓	✓	Ogundipe et al., 2020¶
	NR	Intestine	Flies	275	*nuc* gene PCR, MALDI-TOF	4	CA	–	–	✓	–	–	✓	✓	✓	✓	✓	Onwugamba et al., 2020
	2015–2016	Fomites	Inanimate materials	14	*nuc* gene PCR, MALDI-TOF	3	CA	–	–	✓	–	–	✓	✓	✓	–	–	Shittu et al., 2020b
Rwanda	2013–2014	Clinical samples	Human	138	NR	39	HA	–	–	–	–	✓	–	–	–	–	–	Masaisa et al., 2018
South Africa	2010–2012	Clinical samples	Human	2709	*nuc* gene PCR	1160	HA	–	–	✓	–	✓	✓	–	–	–	–	Perovic et al., 2015
	2015	Nasal, blood, pus, central venous catheter, sputum, wound	Human	NR	VITEK and MALDI-TOF	27	HA	–	–	–	✓	–	–	–	✓	–	✓	Amoako et al., 2016
	2013–2016	Diverse clinical samples	Human	1914	VITEK 2	482	HA [449], CA [33]	–	–	✓	–	✓	✓	–	–	–	–	Perovic et al., 2017
	2013–2014	Sputum	Human	33	MALDI-TOF	17	HA	–	–	✓	✓	✓	✓	–	✓	✓	–	Mahomed et al., 2018
	NR	Nasal and hands swabs, litter, transport truck, carcass, cecal samples, retail point meats	Farm workers, animals, and slaughterhouse environment	145	API Staph kit	12	LA	–	–	✓	–	✓	✓	✓	–	–	✓	Amoako et al., 2019¶
	2013–2016	Blood samples	Human	2164	API Staph/MALDI-TOF	484	HA/CA	–	–	–	–	✓	–	–	✓	–	–	Singh-Moodley et al., 2019
	2015–2017	Blood	Human	199	VITEK 2	54	HA	–	✓	✓	–	✓	✓	–	–	–	–	Abdulgader et al., 2020
	2010–2017	Blood culture	Human	5820	VITEK/MALDI-TOF and *nuc* gene PCR	2019 (subsampled 48 MRSA isolates)	HA/CA	–	–	✓	–	✓	✓	–	–	–	–	Singh-Moodley et al., 2020
Tanzania	2013–2015	Clinical samples	Human	30	NR	10	HA	–	–	–	–	–	✓	✓	✓	✓	✓	Kumburu et al., 2018
	2015	Raw milk	Raw milk	48	*gltB* gene PCR	3	CA	–	–	✓	–	–	–	–	–	–	–	Mohammed et al., 2018
	2014–2015	Nasal swab, wound swab	Human	158	VITEK	10	HA, CA	–	–	✓	–	–	✓	–	✓	–	–	Moremi et al., 2019
Tunisia		Raw meat	Chicken	43	*nuc* gene PCR	2	LA	–	–	✓	✓	✓	✓	–	✓	✓	✓	Chairat et al., 2015
	2013–2014	Milk from mastitic cow	Cows	15	*nuc* gene PCR	3	LA	–	–	✓	–	✓	✓	–	–	✓	✓	Klibi et al., 2018
	2008–2009	Device-related infection, pus, blood, biological fluid	Human	87	NR	32	HA	–	✓	–	✓	✓	–	–	✓	✓	–	Mesrati et al., 2018
Uganda	2013	Animals	Milk samples	41	NR	23	LA	–	–	✓	✓	✓	–	–	✓	✓	–	Asiimwe et al., 2017b
	2013	Nasal swabs	Human	73	NR	48	CA	–	–	✓	✓	✓	–	–	✓	–	–	Asiimwe et al., 2017a
	2011	Nasopharyngeal samples	Children <5 years	144	NR	45	CA	–	–	✓	–	✓	–	–	✓	–	–	Kateete et al., 2019a,b
Zambia	2009–2012	Pus, blood	Human	NR	NR	32	HA	–	–	✓	–	✓	–	–	✓	–	–	Samutela et al., 2017
**Multicentre studies**																		
Angola, Sao Tome and Principe and Cape Verde	2010–2014	Diverse clinical samples and nasal swabs from health care workers and healthy individual	Human	454	*nuc* gene PCR	162	HA	–	–	✓	✓	✓	✓	–	✓	✓	–	Conceição et al., 2015b
Angola-Sao Tome Principe	2010–2014	Nasal swabs	Human	164	NR	29	HA	–	–	✓	✓	✓	✓	–	✓	✓	–	Conceição et al., 2015a
	2017	Hospital surfaces	Environmental samples	23	NR	16	HA	–	–	✓	✓	✓	✓	–	✓	–	–	Aires-de-Sousa et al., 2018
	2017	Nasal swabs	Human	110	*nuc* gene PCR	33	HA/CA	–	–	✓	✓	✓	✓	–	✓		–	Rodrigues et al., 2018
Cameroon-South Africa	2016	Nasal and rectal swabs and hand swabs from human	Pigs/human	NR	VITEK 2	5	LA	–	–	✓	–	✓	✓	✓	✓	✓	✓	Founou et al., 2019¶
DR Congo-Cote d’Ivoire	2010–2013	Nares swabs	Human and animals	495	*nuc* gene PCR	19	HA/LA	–	–	✓	–	–	✓	–	✓	✓	–	Schaumburg et al., 2015

NR, not reported; HA, hospital-associated; CA, community-associated; LA, livestock-associated; –, not determined; *, other toxin/virulence associated genes; ¶, studies that provided sufficient genotyping data based on whole genome sequencing (WGS).

Identification of *S. aureus* in more than 50% (36/65) of the eligible studies was based on protein profiling (MALDI-TOF) or methods established on PCR detection of conserved (16S rRNA, *nuc*, *tuf*, *gltB*) genes, or the combination of both. The detection of antibiotic resistance and toxin/virulence genes were described only in 37% (24/65) and 83% (54/65) of the studies, respectively (Table 1). One study reported *mecC*-positive MRSA from animals (Dweba and Zishiri, 2019). While all the eligible studies characterized MRSA using at least one molecular typing technique, only 40% (26/65) from 16 African countries provided sufficient genotyping data (Supplementary Table 3). Furthermore, 12 studies performed whole-genome sequencing (WGS), of which eight carried out adequate analyses to infer MRSA clones (Table 1).

### Source of Methicillin-Resistant *Staphylococcus aureus*

Methicillin-resistant *Staphylococcus aureus* from the eligible studies was classified as either HA, CA, or livestock-associated (LA) based on their source of isolation as provided in the articles. Overall, 40% (26/65) of the studies were on HA-MRSA, while 18% (12/65) each were from the community and animal/livestock settings (Table 1). Additionally, 22% (14/65) of studies characterized MRSA from either two (HA-CA: *n* = 10; HA-LA: *n* = 2; CA-LA: *n* = 1) or all the study settings (HA-CA-LA: *n* = 1). We could not infer the source of isolates in one study.

### High Clonal Diversity Among Methicillin-Resistant *Staphylococcus aureus* Isolates Reported in Africa

**FIGURE 2:**
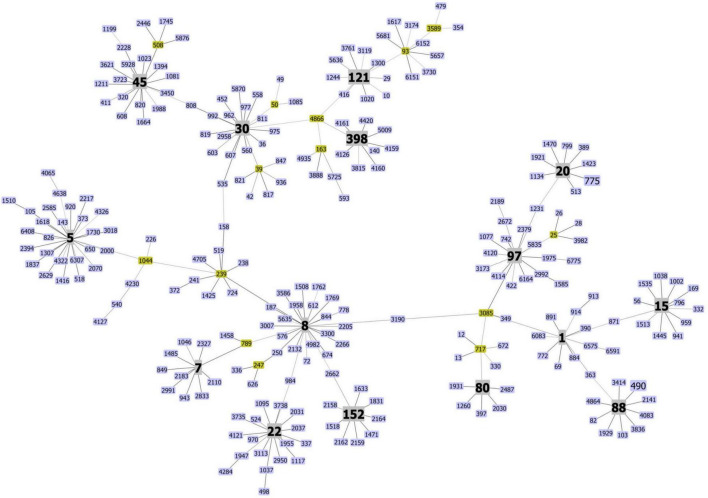
Clonal diversity of methicillin-resistant *Staphylococcus aureus* in Africa. The minimum spanning tree was constructed with Phyloviz software version 2.0 hosted on http://www.phyloviz.net. The allelic profiles were obtained from the MLST database hosted on (https://pubmlst.org/organisms/staphylococcus-aureus) that included the sequence types of the MRSA described in this review, and 236 randomly selected STs based on the differences in their allelic profiles and representative of the MRSA diversity worldwide. Each node depicts an ST, and nodes centrally located and bearing different colors correspond to a group founder or sub-founder. Clonal complexes (CCs) reported in this study are colored in gray.

**FIGURE 3:**
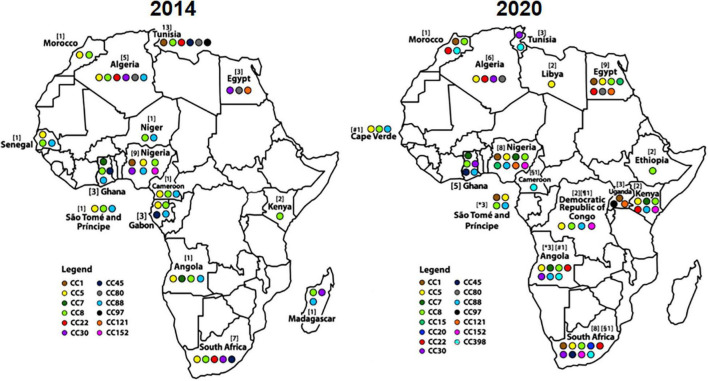
Distribution of methicillin-resistant *Staphylococcus aureus* (MRSA) clones in Africa as reported (Abdulgader et al., 2015) and this current study (2020). Each clonal complex (CC) is represented with colored oval shape and eligible studies carried out in each country is indicated in parenthesis. The symbols (#, *, §, or ¶) on the right side (2020) depict data that were extracted from the same multicentre study.

We observed a high genetic heterogeneity among MRSA in the 26 eligible studies that provided sufficient genotyping data. Based on MLST, they were classified into 39 STs, four of which were unassigned types (Supplementary Table 3). The MLST cluster analysis using Phyloviz based on the geoBURST algorithm revealed 15 CCs. They comprised mainly CC1, CC5, CC8, CC22, CC30, and CC88. Others were CC7, CC15, CC20, CC45, CC80, CC97, CC121, CC152, and CC398 (Figures 2, 3).

### Clonal Complex 1

This clone was identified in six countries (Figure 3). PVL-positive t590-ST1-V [5C2] was documented from nasal samples both in hospitalized patients and health care workers (HCWs) in São Tomé and Príncipe (Conceição et al., 2015a,b). Another PVL-positive lineage: t657-ST772-V [5C2] (Bengal Bay Clone), was detected from human nasal samples in the community setting in Nigeria (Ogundipe et al., 2020). Moreover, PVL-negative t127-ST1-IV [2B] was described in a nasal sample of a non-hospitalized individual in Morocco (Mourabit et al., 2017), while it’s variant (t127-ST1-V [5C2]) was identified from non-human specimens (milk products) in Uganda (Asiimwe et al., 2017b). ST1-V [5C2] and ST913-V [5C2] were recovered from clinical samples in Egypt (Soliman et al., 2020). In South Africa, t465-ST1-I [1B]/IV [2B] was isolated from patients with cystic fibrosis (CF) (Mahomed et al., 2018).

### Clonal Complex 5

This lineage was reported in 10 countries (Figure 3). The PVL-negative t105-ST5-IV [2B] was the dominant lineage colonizing patients and HCWs (Conceição et al., 2015b; Rodrigues et al., 2018), as well as inanimate surfaces in Angola (Aires-de-Sousa et al., 2018). Also, it was detected in nasal samples of patients and HCWs in São Tomé and Príncipe (Conceição et al., 2015b), and a community patient admitted to a hospital in Algeria (Djoudi et al., 2014). In the DRC, three ST5-IV [2B] variants (t002-ST5-IV [2B], t105-ST5-IV [2B], and PVL-positive t311-ST5-IV [2B]) were described (Lebughe et al., 2017; Vandendriessche et al., 2017). In Kenya, t13150-ST5-II [2A] and t007-ST39-II [2A] were identified from clinical samples (Omuse et al., 2016; Kyany’a et al., 2019). ST5-VI [4B] was reported in a tertiary care hospital in Egypt (Soliman et al., 2020) and Cape Verde (Conceição et al., 2015b). ST5-VII [5C1] was recovered from a patient in the nephrology ward in Algeria (Djoudi et al., 2014). Other reports include ST5-III/V/non-typeable (NT) in South Africa (Abdulgader et al., 2020; Singh-Moodley et al., 2020). The related genotypes such as t6065-ST5/ST2629-V [5C2] in Angola (Conceição et al., 2015a,b), t6065-ST69-V [5C2] in Libya (Khemiri et al., 2017), and t002-ST105-II [2A] in São Tomé and Príncipe (Conceição et al., 2015b) were also noted. One study reported t002/t11469-ST5-V [5C2] in poultry birds (Nworie et al., 2017) in Nigeria (Supplementary Table 3).

### Clonal Complex 8

ST8-IV [2B] (with diverse *spa* types) was documented in hospitalized patients and HCWs in Angola, Cape Verde, and São Tomé and Príncipe (Conceição et al., 2015a; Rodrigues et al., 2018), and clinical samples in Ghana (Egyir et al., 2015) and Kenya (Omuse et al., 2016). PVL-positive t121-ST8-IV [2B] was identified in Cape Verde (Conceição et al., 2015b), Ghana (Egyir et al., 2015), and São Tomé and Príncipe (Rodrigues et al., 2018). The t451-ST8-V [5C2] was one of the dominant clones among hospitalized patients and HCWs in São Tomé and Príncipe (Conceição et al., 2015a,b; Rodrigues et al., 2018). Also, ST8-V [5C2] was described in hospital settings in Angola (Conceição et al., 2015b; Aires-de-Sousa et al., 2018), Egypt (Soliman et al., 2020), Ghana (Egyir et al., 2015), and Kenya (Omuse et al., 2016). The PVL-negative ST8-V/VII (largely t1476) was the major clone in the DRC (Lebughe et al., 2017; Vandendriessche et al., 2017), and Angola (Aires-de-Sousa et al., 2018). Two countries, i.e., Morocco (Mourabit et al., 2017) and Nigeria (Ogundipe et al., 2020), described ST8-V [5C2] with different *spa* types (t2231, t2658, and t12236) in non-clinical settings. The t456-ST8-I [1B] was only identified in South Africa (Mahomed et al., 2018). Furthermore, ST239/ST241-III [3A] was noted in hospital settings in Egypt (Soliman et al., 2020), Ghana (Egyir et al., 2015), Kenya (Omuse et al., 2016; Kyany’a et al., 2019), and South Africa (Abdulgader et al., 2020; Singh-Moodley et al., 2020). ST612-IV [2B], which comprised mainly *spa* type t1257, was a major clone in clinical (Singh-Moodley et al., 2020) and non-clinical settings (Amoako et al., 2019) in South Africa. Other related STs include ST72-V [5C2] in Angola (Conceição et al., 2015b; Aires-de-Sousa et al., 2018; Rodrigues et al., 2018) and ST4705-III [3A] in Kenya (Kyany’a et al., 2019).

### Clonal Complex 22

ST22-MRSA was identified in six African countries. They include Angola (Conceição et al., 2015b), Algeria (Djoudi et al., 2014), Egypt (Soliman et al., 2020), Kenya (Omuse et al., 2016), and South Africa (Abdulgader et al., 2020; Singh-Moodley et al., 2020). Various *spa* types (t005, t012, t022, t032, t223, t6397, t11293, and t13149) were associated with this lineage that harbored the SCC*mec* IV element (Supplementary Table 3). Moreover, it was the major clone recovered from nasal samples of volunteers and outpatients in Tangier, Morocco (Mourabit et al., 2017). Most MRSA isolates from Algeria and Morocco possessed the gene encoding for toxic shock syndrome (*tst*).

### Clonal Complex 30

This clone was observed in both human and animal samples. We identified seven *spa* types (t012, t018, t030, t037, t045, t064, and t6278; Supplementary Table 3). In South Africa, ST30-II [2A], ST36-II [2A], and ST36-III [3A] were identified from bacteremic patients (Abdulgader et al., 2020; Singh-Moodley et al., 2020), including ST30-I/IV [2B] from CF patients (Mahomed et al., 2018). ST30-V [5C2] was reported in different settings in Angola (Conceição et al., 2015a,b; Rodrigues et al., 2018), and from a chicken meat sample in Tunisia (Chairat et al., 2015). One isolate characterized as t018-ST36-II [2A] was described in Ghana (Egyir et al., 2015) and from the rinsate of processed animals in an abattoir in South Africa (Amoako et al., 2019). Furthermore, the genetically related ST535-IV [2B] was described in a patient in a nephrology ward in Algeria (Djoudi et al., 2014).

### Clonal Complex 88

ST88-IV [2B] with diverse *spa* types (t186, t325, t335, t786, t1451, t1603, t1814, t3869, and t12827) was documented in eight studies from seven African countries (Supplementary Table 3 and Figure 3), particularly in Portuguese-speaking nations. It was widely distributed in Angola (Conceição et al., 2015a,b; Aires-de-Sousa et al., 2018; Rodrigues et al., 2018), Cape Verde (Conceição et al., 2015b), and São Tomé and Príncipe (Conceição et al., 2015a,b; Aires-de-Sousa et al., 2018; Rodrigues et al., 2018). Other reports include the DRC (Lebughe et al., 2017; Vandendriessche et al., 2017), and Ghana (Egyir et al., 2015; Wolters et al., 2020). PVL-negative ST88-IV [2B] was recovered from nasal samples of both humans and pigs in Nigeria (Otalu et al., 2018), and ST88-V [5C2] was detected in a blood culture sample in the DRC (Vandendriessche et al., 2017). ST88-MRSA with a NT SCC*mec* was identified in Kenya (Omuse et al., 2016).

### Other Clonal Complexes

These include eight clones that belonged to smaller (in number or limited spread across countries) groups (Supplementary Table 3 and Figure 3). They consist of CC7 (ST789-IV [2B]/V [5C2]) (Egyir et al., 2015; Omuse et al., 2016; Ogundipe et al., 2020), CC15 (ST15-V [5C2], and ST1535-V [5C2]) (Nworie et al., 2017; Soliman et al., 2020), and CC20 (ST20-IV [2B]) (Mahomed et al., 2018). CC45 comprising ST45-I [1B], ST45-IV [2B], and ST508-I [1B] was detected in CF patients in South Africa (Mahomed et al., 2018). Also, ST508-V [5C2] associated with CC45 was described in Ghana (Egyir et al., 2015). PVL-positive CC80 (ST80-IV [2B]) was only described in Algeria (Djoudi et al., 2014; Agabou et al., 2017) and Egypt (Soliman et al., 2020). CC152 (mostly PVL-positive) with various *spa* types (t355, t715, t4960, t5691, and t15644) and SCC*mec* types (I, II, IV, V, and VII) were identified in four countries. They include the DRC (Lebughe et al., 2017; Vandendriessche et al., 2017), Kenya (Kyany’a et al., 2019), Nigeria (Ogundipe et al., 2020), and South Africa (Mahomed et al., 2018). ST121-V [5C2] was documented in Egypt (Soliman et al., 2020) and Uganda (Asiimwe et al., 2017b), in addition to PVL-positive isolates in Nigeria (Ogundipe et al., 2020). The LA ST398-IV [2B]/V [5C2] was recovered from the rectal and nasal samples of pigs in Cameroon, South Africa (Founou et al., 2019), and in the nasal sample of a healthy individual in Morocco (Mourabit et al., 2017). Also, ST398-IV [2B] was detected in raw meat samples in Tunisia (Chairat et al., 2015). MRSA with the genotype ST140-IV [2B] (associated with CC398) was recovered from inanimate surfaces in a health care institution in Angola (Aires-de-Sousa et al., 2018).

### The Dynamics of Methicillin-Resistant *Staphylococcus aureus* Clones (2014–2020)

We compared MRSA clones reported from a previous study (Abdulgader et al., 2015) and the period under review. New genotyping data were available from Cape Verde, Ethiopia, the DRC, Libya, and Uganda. However, reports on MRSA clones from Senegal, Gabon, and Madagascar in the previous study were absent in the current period under review. Overall, genotyping data from 11 African countries (Angola, Algeria, Cameroon, Egypt, Ghana, Kenya, Tunisia, Morocco, Nigeria, São Tomé and Príncipe, and South Africa) in the two study periods were identified and compared (Figure 3). We observed an increase in the number of MRSA clones reported in seven (Angola, Egypt, Kenya, Morocco, Nigeria, São Tomé and Príncipe, and South Africa) of the 11 countries. Specifically, CC1, previously described only in Nigeria and Tunisia (Abdulgader et al., 2015), was identified in clinical and non-clinical settings in six countries (Egypt, Morocco, Nigeria, São Tomé and Príncipe, South Africa, and Uganda). ST22-IV [2B] (CC22), previously documented only in South Africa (Abdulgader et al., 2015), was described in Angola, Algeria, Egypt, Kenya, Morocco, and South Africa. Also, CC152-MRSA identified only in Nigeria (Abdulgader et al., 2015) was reported in the DRC, Kenya, Nigeria, and South Africa. In contrast, the HA Brazilian/Hungarian clone (ST239/241-III [3A]), which was previously described as a major clone on the continent, was noted only in four countries (Egypt, Ghana, Kenya, and South Africa). Some MRSA clones were still limited to specific countries and regions. ST80-IV [2B] (CC80) and ST612-IV [2B] (CC8) were identified only in North African countries and South Africa, respectively. Overall, CC5-MRSA, CC8-MRSA, and CC88-MRSA remained widely distributed across the continent (Figure 3).

### Panton–Valentine Leukocidin-Positive Methicillin-Resistant *Staphylococcus aureus* and Clonal Population in Africa

Methicillin-resistant *Staphylococcus aureus* carriage of the PVL gene was investigated in 50 of the 65 eligible studies. PVL-positive isolates were reported in 26 studies in 11 countries (Table 1 and Supplementary Table 3). The lineages and countries were: CC1 (Egypt, São Tomé and Príncipe, and Nigeria), CC5 (Algeria, Angola, and DRC), CC8 (Cape Verde, DRC, Ghana, São Tomé and Príncipe, and South Africa), CC22 (Angola), and CC30 (Angola and South Africa). Others are CC80 (Algeria and Egypt), CC121 (Nigeria, Egypt, and Uganda), CC152 (DRC and Nigeria), and CC398 (Cameroon). The prevalence of PVL-positive MRSA ranged from 0% (Otalu et al., 2018) to 77% (23/30) (Ogundipe et al., 2020), with a median of 33% (Table 2).

**TABLE 2:** Prevalence of Panton–Valentine leukocidin (PVL) gene reported in eligible studies with ≥30 methicillin-resistant *Staphylococcus aureus* (MRSA) isolates.

Country	No of MRSA	No of PVL-positive MRSA	% Prevalence	References
Angola	127	2	2	Conceição et al., 2015b
DR Congo	55	5	9	Lebughe et al., 2017
	27	2	7	Vandendriessche et al., 2017
	30	22	73	Abd El-Hamid and Bendary, 2015
	34	5	15	Khairalla et al., 2017
	65	30	46	Abd El-Hamid et al., 2019
	120	40	33	Shehata et al., 2019
Libya	95	32	34	Ahmed et al., 2017
Nigeria	66	6	9	Alli et al., 2015
	38	0	0	Otalu et al., 2018
	30	23	77	Ogundipe et al., 2020
South Africa	484 (subsampled 108 MRSA isolates)	27	25	Singh-Moodley et al., 2019
Uganda	48	25	52	Asiimwe et al., 2017a
	45	19	42	Kateete et al., 2019a
Zambia	32	3	9	Samutela et al., 2017

## Discussion

This systematic review provided an update on the diversity of MRSA clones in Africa for the past 6 years (2014–2020). We observed a slight increase in the number of studies and countries that provided sufficient genotyping data. Diverse MRSA clones were distributed across human, environmental, and animal settings. CC5, CC8, and CC88 were the major clones identified in Africa. Various *spa* types and SCC*mec* elements characterized CC5-MRSA. It was postulated that the African ST5-MRSA evolved from ST5-MSSA through acquiring the SCC*mec* element (Schaumburg et al., 2014). Its capacity and higher propensity to acquire various SCC*mec* elements could play a significant role in its increased dissemination and adaptation to different environments in Africa. However, the phylogeny, origin, and features for the spread of CC5-MRSA remain unclear in Africa.

Five SCC*mec* types and 11 *spa* types were associated with CC8-MRSA suggesting its high diversity in Africa. The CC8 is comprised of the hospital (Archaic [ST250], Iberian [ST247], and Brazilian/Hungarian/EMRSA-1 [ST239]) and CA (USA300 [t008-ST8], USA500 [t064-ST8]) clones (Bowers et al., 2018). ST239 was described as a major clone on the continent (Abdulgader et al., 2015) but has declined in the current period under review. It was identified only in four countries. ST239-MRSA evolved from recombination events between ST8 and ST30, in addition to the acquisition of antibiotic resistance and virulence determinants that contribute to its pathogenic capabilities (Robinson and Enright, 2004; Gill et al., 2021). However, this clone’s low competitive potential relative to ST8 and ST30 could contribute to its gradual decline in different continents (Dai et al., 2019; Gill et al., 2021).

USA300 isolates harbor the SCC*mec* type IVa element, PVL-positive, with the arginine catabolic mobile element (ACME). These factors are lacking in USA500 isolates. A phylogenomic study provided some insights on the origin and the features for the spread of ST8-MRSA in Africa (Strauß et al., 2017). First, the heterogeneity of SCC*mec* types suggests the different introduction of these genetic elements to the ST8 genetic background. Secondly, African USA300 isolates formed a monophyletic group within the North American Epidemic (NAE) USA300 clade. This observation suggests a single introduction episode of this clone to the African continent followed by an extensive spread in the population (Strauß et al., 2017). However, it should be noted that the African USA300 isolates analyzed in the investigation were PVL-positive, unlike most of the MRSA (PVL-negative) identified in our study (Supplementary Table 3). Also, a phylogenetic analysis of t1476-ST8-IV-MRSA isolates (PVL, ACME-negative) from HIV-infected patients in Tanzania (Manyahi et al., 2021) revealed that they were unrelated to NAE USA300 and the African USA300 previously described in Gabon and East Africa. We hypothesize that t1476-ST8-MRSA from Tanzania, Angola, DRC, and Kenya (Supplementary Table 3) may have acquired different SCC*mec* elements despite sharing common genetic characteristics. Further studies are needed to unravel the origin and nature of CC8-MRSA in Africa.

CC88-MRSA is regarded as an “African” clone due to its wide distribution in West, Central, and East Africa (Schaumburg et al., 2014). It is noteworthy that CC88-MRSA was widely distributed in Portuguese-speaking African countries (Angola, Cape Verde, and São Tomé and Príncipe). The reasons for this observation are unclear. However, we postulate that demographic and cultural relationships could play a significant role in establishing this clone in these African countries. We observed an expansion of CC1-MRSA, CC22-MRSA, and CC152-MRSA in Africa. Unlike the European CC1-MRSA, which is mainly t127-ST1-IV [2A], the African CC1-MRSA (identified in six countries) comprised *spa* types t127, t465, and t590, and most of them harbored the SCC*mec* V element. ST22-IV [2B] (CC22), which is tagged epidemic MRSA-15 (EMRSA-15), was previously documented only in South Africa (Abdulgader et al., 2015), but now in six African countries. The CC152 lineage is a successful MSSA clone in Africa that is mainly PVL-positive. CC152-MRSA was previously noted in Nigeria (Abdulgader et al., 2015), but it is now described in four countries. The increasing trend of CC152-MRSA with diverse *spa* types and SCC*mec* elements in Africa is also noteworthy. This observation supports the evidence of multiple introductions among MSSA isolates in sub-Saharan Africa as the basis for the evolution of this clone (Baig et al., 2020). Recent studies have also reported CC152-MRSA from humans (Egyir et al., 2020, 2021) and animals (Shittu et al., 2021), including fomites (Shittu et al., 2020b) in Africa. The emergence of PVL-positive CC152-MRSA is of public health concern. Hence, there is a need to understand the dynamics for introducing and acquiring the *mecA* gene by CC152-MSSA isolates in Africa.

ST80-IV [2B] (CC80) was limited to North African countries and ST612-IV [2B] (CC8) in South Africa, as described previously (Abdulgader et al., 2015). However, MRSA in various STs (ST80, ST728, ST1931, ST2030, ST3247, and ST5440) assigned to CC80 was recently described in environmental samples associated with livestock in South Africa (Ramaite et al., 2021). ST612-IV [2B] has been detected in wound patients in Tanzania (Moremi et al., 2019). Also, it has been described in a poultry farm and workers in South Africa, raising concerns about its spread across the poultry food chain (Amoako et al., 2019). There is still a paucity of data on the molecular epidemiology of MRSA in animals in Africa. Hence, their role in the dissemination of MRSA remains unclear. Nonetheless, we observed diverse clones (ST1, ST5, ST8, ST36, and ST88) with various SCC*mec* types associated with the hospital and community settings recovered from livestock and their surroundings. Our findings suggest human to animal transmission and adaptation in poultry and food animals, which warrants further investigations. These observations somewhat indicate the changing epidemiological landscape and highlight the need for a “One-Health” approach to understanding MRSA epidemiology in Africa.

Panton–Valentine leukocidin is a pore-forming protein consisting of two sub-units (lukF-PV, lukS-PV) that target human granulocytes, monocytes, and macrophages (Holzinger et al., 2012). It is mainly associated with skin and soft tissue infection (SSTI) (Friesen et al., 2020), and in particular, pyomyositis in developing countries (Shittu et al., 2020a). This study identified PVL-positive MRSA from nine CCs in 10 countries. Africa is regarded as a PVL-endemic region (Schaumburg et al., 2015). The high prevalence (median: 33%) of PVL-positive MRSA, particularly among nasal samples of hospitalized patients and non-hospitalized individuals in Africa (Supplementary Table 3), is of public health concern. Recurrent SSTIs are associated with *S. aureus* carriers colonized with PVL-positive *S. aureus* (Rentinck et al., 2021). So far, the burden of PVL-positive *S. aureus* is not well known despite its high prevalence in Africa. Knowledge on factors that contribute to the high prevalence of PVL in Africa could help unravel the pathogenic role of PVL and develop strategies against PVL-related diseases.

Genomic epidemiology is a powerful tool to provide valuable information on the emergence of high-risk pandemic clones, antibiotic resistance mechanisms, and virulence determinants (Baker et al., 2018). The characterization of MRSA using conventional molecular typing techniques (e.g., *spa* typing, MLST) describes only a fraction of the entire *S. aureus* genome (Price et al., 2013). WGS offers a better opportunity to expand our knowledge about clinical and epidemiologic aspects of MRSA infection and colonization, including transmission patterns, evolution, and guide on appropriate interventions (Humphreys and Coleman, 2019). Our data showed that 12 of the 26 studies utilized WGS. However, eight provided sufficient genotyping data. Understanding the epidemiology of MRSA based on WGS is still in its infancy in Africa. Nonetheless, international scientific cooperation efforts support genomic sequencing capacity building on the continent, e.g., the Fleming Fund, SEQAFRICA. It is expected that these initiatives will provide quality genotyping data that will assist in MRSA surveillance in Africa. However, these efforts will require complementary local investment to ensure quality and representative genotyping data and sustainability.

In August 2017, two independent consortia converged to form the StaphNet Africa. This consortium was co-convened by the corresponding author and Dr. Beverly Egyir (Ghana). The first kick-off meeting took place at the Noguchi Memorial Institute for Medical Research, University of Ghana. The conference, sponsored by the Wellcome Trust-Cambridge Centre for Global Health Research, brought together biomedical scientists and physicians with a research focus on *S. aureus* from 10 African countries (Nigeria, Ghana, Egypt, Gabon, Kenya, Mozambique, South Africa, Uganda, Kenya, and the Gambia), and the United Kingdom. Although the network’s activities have been hampered by funding, one of its resolutions was to provide regular updates on the epidemiology of *S. aureus* in Africa. This systematic review is an affirmation of this resolution. Also, an African version of the biennial International Symposium on Staphylococci and Staphylococcal Infections (ISSSI), known as the African Symposium on Staphylococci and Staphylococcal Infections (ASSSI), was adopted for implementation. The symposium is to provide a platform for researchers to network and share current research work on *S. aureus* in Africa. We anticipate that this initiative, with others, will provide periodic data on MRSA surveillance in Africa.

## Conclusion

We have provided an update on the clonal diversity of MRSA in Africa in the past 6 years. Nonetheless, there is still a paucity of data as sufficient genotyping data were available in only 16 of 54 (30%) countries. This systematic review did not investigate antibiotic resistance and virulence gene repertoire of the various African MRSA clones and their level of transmissibility. The origin and features underlying the spread of MRSA clones in Africa are not clear. Identifying human-associated lineages in food animals and products provides evidence to adopt a “One-Health” approach to understand the epidemiology of MRSA in Africa. There is a need to develop robust local capacity in genotyping, including WGS technologies, to determine the genetic factors that contribute to the evolution and adaptation of various African MRSA clones. Lastly, an active continent-wide antimicrobial resistance surveillance program and data exchange across One-Health sectors and professionals are required to monitor the clonal dissemination and emergence of new MRSA clones in Africa.

## Author Contributions

AS, BE, SA, and AA initiated the update on the diversity of MRSA in Africa. OL retrieved the data from the various databases and was jointly reviewed by AS. AS and OL wrote the initial manuscript. All authors reviewed and agreed on the final version of the manuscript before submission for peer review.

## Conflict of Interest

The authors declare that the research was conducted in the absence of any commercial or financial relationships that could be construed as a potential conflict of interest.

## Publisher’s Note

All claims expressed in this article are solely those of the authors and do not necessarily represent those of their affiliated organizations, or those of the publisher, the editors and the reviewers. Any product that may be evaluated in this article, or claim that may be made by its manufacturer, is not guaranteed or endorsed by the publisher.

## Footnotes

1
http://www.phyloviz.net

2
https://pubmlst.org/organisms/staphylococcus-aureus

## Supplementary Material

Supplementary Table 1 Search strings used to identify eligible studies available in six electronic databases.

Supplementary Table 2 Sequence types and corresponding allele profiles used for clustering analysis.

Supplementary Table 3 Summary of the methicillin-resistant *Staphylococcus aureus* (MRSA) clones reported in 26 eligible studies.
