# Supplementary material for: A 6-Year Update on the Diversity of Methicillin-Resistant Staphylococcus aureus Clones in Africa: A Systematic Review
Source: Front Microbiol. 2022 May 3;13:860436. doi: 10.3389/fmicb.2022.860436 (PMC9113548; doi:10.3389/fmicb.2022.860436)
Supplement: Supplementary Table 1 — Search strings used to identify eligible studies available in six electronic databases. [file Table_1.DOC]

| **Supplementary Table 1** | | |
| --- | --- | --- |
| **Database** | **Search period** | **Search terms** |
| MEDLINE via PubMed | November 2014 - December 2020 | (MRSA or Methicillin Resistant *Staphylococcus aureus*) OR (Livestock OR Community-and Hospital-Acquired MRSA or Methicillin Resistant *Staphylococcus aureus*) OR (Occurrence of MRSA OR Methicillin Resistant *Staphylococcus aureus*) OR (Prevalence of MRSA or Methicillin Resistant *Staphylococcus aureus*) OR (Molecular epidemiology of MRSA or Methicillin Resistant *Staphylococcus aureus*) OR (Outbreak of MRSA or Methicillin Resistant *Staphylococcus aureus*)  AND  ("Africa" OR "Algeria" OR "Angola" OR "Benin" OR "Benin Republic" OR "Botswana" OR "Burkina Faso" OR "Burundi" OR "Cabo Verde" OR "Cameroon" OR "Central African Republic" OR "Chad" OR "Comoros" OR "Democratic Republic of Congo" OR "Cote d'Ivoire" OR Ivory coast” OR "Djibouti" OR "Egypt" OR "Equatorial Guinea" OR "Eritrea" OR "Eswatini" OR "Swaziland" OR "Ethiopia" OR "Gabon" OR "Gambia" OR "Ghana" OR "Guinea" OR "Guinea-Bissau" OR "Kenya" OR "Lesotho" OR "Liberia" OR "Libya" OR "Madagascar" OR "Malawi" OR "Mali" OR "Mauritania" OR "Mauritius" OR "Morocco" OR "Mozambique" Mozambique OR “Moçambique” OR "Namibia" OR "Niger" OR "Nigeria" OR "Rwanda" OR "Sao Tome and Principe" OR "Senegal" OR "Seychelles" OR "Sierra Leone" OR "Somalia" OR "South Africa" OR "South Sudan" OR "Sudan" OR "Tanzania" OR "Togo" OR "Tunisia" OR "Uganda" OR "Zambia" OR "Zimbabwe") |
| EBSCOhost via Academic Search premier, Africa-Wide information and CINAHL |
| ISI Web of Science |
| African Journals Online (AJOL) |
| Scopus |
| Google Scholar | November 2014 - December 2020 | (MRSA OR Methicillin Resistant *Staphylococcus aureus*) AND  "Africa" |
|
| ¶Publish or Perish |

¶A literature and citation mining algorithm (Harzing, 2007)
