# Supplementary material for: A 6-Year Update on the Diversity of Methicillin-Resistant Staphylococcus aureus Clones in Africa: A Systematic Review
Source: Front Microbiol. 2022 May 3;13:860436. doi: 10.3389/fmicb.2022.860436 (PMC9113548; doi:10.3389/fmicb.2022.860436)
Supplement: Supplementary Table 3 — Summary of the methicillin-resistant Staphylococcus aureus (MRSA) clones reported in 26 eligible studies. [file Table_3.DOC]

**Supplementary Table 3: Summary of the Methicillin-resistant *Staphylococcus aureus* (MRSA) clones reported in 26 eligible studies.**

| **Country** | **No of MRSA** | **Sample** | **Setting** | **ST-SCC*mec*** | ***spa* type** | **MLST Clonal complex** | **PVL** | **Reference** |
| --- | --- | --- | --- | --- | --- | --- | --- | --- |
| Algeria | 6 | Nasal | LA | ST80-IV [2B] | ND | CC80 | + | Agabo*u et* al., 2017 |
|  | 9 | Nasal | HA | ST5-IVc [2B] | ND | CC5 | - | Djoud*i et* al., 2014 |
|  |  |  |  | ST5-VII [5C1] | ND | CC5 | - |  |
|  |  |  |  | ST22-IVa [2B] | t223 | CC22 | - |  |
|  |  |  |  | ST535-IVh [2B] | ND | CC30 | - |  |
|  |  |  |  | **ST80-IVa/IVc [2B]** | ND | CC80 | + |  |
|  |  |  |  | ST80-IVa [2B] | ND | CC80 | - |  |
| Angola | 9 | Nasal | HA | ST5/ST2629-V [5C2] | t6065 | CC5 | - | Conceicao *et al* 2015a |
|  |  |  |  | ST8-VII [5C1] | t1476 | CC8 | - |  |
|  |  |  |  | ST30-V [5C2] | t6278 | CC30 | - |  |
|  |  |  |  | **ST88-IVa [2B]** | t186, t325, t786, t1951 | CC88 | - |  |
|  |  |  |  | **ST5-IVa [2B]** | **t105**, t311, t11657 | CC5 | - |  |
|  | 127 | Nasal | HA | ST5/ST2629-V [5C2] | t6065 | CC5 | - | Conceicao *et al* 2015b |
|  |  |  |  | ST8-IVc/IVd/V | t064, t104, t1771 | CC8 | - |  |
|  |  |  |  | ST8-VII [5C1] | t1476 | CC8 | - |  |
|  |  |  |  | ST72-V [5C2] | t148, t3092 | CC8 | - |  |
|  |  |  |  | ST22-IVc [2B] | t005 | CC22 | + |  |
|  |  |  |  | ST30-V [5C2] | t6278 | CC30 | + |  |
|  |  |  |  | ST88-IVa [2B] | t186, t325, t786, t1951, t3869 | CC88 | - |  |
|  |  |  |  | **ST5-IVa [2B]** | **t105** | CC5 | - |  |
|  | 24 | Nasal | CA/HA | **ST5-IVa [2B]** | **t105** | CC5 | - | Rodrigue*s et* al., 2018 |
|  |  |  |  | ST8-VII [5C1] | t1476 | CC8 | - |  |
|  |  |  |  | ST72-V [5C2] | t148 | CC8 | - |  |
|  |  |  |  | ST72-V [5C2] | t3092 | CC8 | - |  |
|  |  |  |  | ST30-V [5C2] | t6278 | CC30 | + |  |
|  |  |  |  | ST88-IVa [2B] | t325/t786 | CC88 | - |  |
|  | 12 | Environmental surfaces | HA | **ST5-IVa [2B]** | **t105** | CC5 | - | Aires-de-Sous*a et* al., 2018 |
|  |  |  |  | ST8-V [5C2] | t1476 | CC8 | - |  |
|  |  |  |  | ST72-V [5C2] | t148 | CC8 | - |  |
|  |  |  |  | ST88-IVa [2B] | t335/t786 | CC88 | - |  |
|  |  |  |  | ST140-IVg [2B] | t957 | CC398 |  |  |
| Cameroon | 1 | Rectal | LA | ST398-Vc [5C2] | t011 | CC398 | + | Founo*u et* al., 2019 |
| Cape Verde | 6 | Nasal | HA | ST8-IVa [2B] | t121 | CC8 | + | Conceiçã*o et* al., 2015b |
|  |  |  |  | ST88-IVa [2B] | t186, t12827 | CC88 | - |  |
|  |  |  |  |  |  |  |  |  |

| **Country** | **No of MRSA** | **Sample** | **Setting** | **ST-SCC*mec*** | ***spa* type** | | **MLST Clonal complex** | **PVL** | **Reference** |
| --- | --- | --- | --- | --- | --- | --- | --- | --- | --- |
| DR Congo | 55 | Nasal/clinical | HA | ST5-IV [2B] | t002, t105 | | CC5 | - | Lebugh*e et* al., 2017 |
|  |  |  |  | ST5-IV [2B] | t311 | | CC5 | + |  |
|  |  |  |  | **ST5-VI [4B]** | t002 | | CC5 | **-** |  |
|  |  |  |  | **ST8-V/VII [5C]** | t1476, t6940, t12377, t15642, t15643 | | CC8 | - |  |
|  |  |  |  | ST8-V/VII [5C] | t1476 | | CC8 | + |  |
|  |  |  |  | ST88-IV [2B] | t690, t786, t4013 | | CC88 | - |  |
|  |  |  |  | ST152-V/VII [5C] | t5691, t15644 | | CC152 | + |  |
|  | 27 | Blood culture | HA | ST5-IV [2B] | t311 | | CC5 | + | Vandendriessch*e et* al., 2017 |
|  |  |  |  | ST8-V [5C2] | t1476 | | CC8 | - |  |
|  |  |  |  | ST88-IV [2B] | t186 | | CC88 | - |  |
|  |  |  |  | ST88-V [5C2] | t186 | | CC88 | - |  |
|  |  |  |  | ST152-V [5C2] | t5691 | | CC152 | + |  |
| Egypt | 18 | Clinical | HA | ST1-V [5C2] | ND | | CC1 | + | Solima*n et* al., 2020 |
|  |  |  |  | ST1-V [5C2] | ND | | CC1 | - |  |
|  |  |  |  | ST913-V [5C2] | ND | | CC1 | + |  |
|  |  |  |  | ST5-VI [4B] | ND | | CC5 | - |  |
|  |  |  |  | ST8-V [5C2] | ND | | CC8 | - |  |
|  |  |  |  | ST8-NT | ND | | CC8 | - |  |
|  |  |  |  | ST239-III [3A] | ND | | CC8 | - |  |
|  |  |  |  | **ST1535-V [5C2]** | ND | | CC15 | - |  |
|  |  |  |  | ST22-IVa [2B] | ND | | CC22 | - |  |
|  |  |  |  | ST80-IVc [2B] | ND | | CC80 | + |  |
|  |  |  |  | ST121-V [5C2] | ND | | CC121 | + |  |
| Ethiopia | 1 | Clinical | HA | ST5635-IVa [2B] | ND | | CC8 | - | Verdú-Expósit*o et* al., 2020 |
| Ghana | 24 | Clinical, nasal swab | CA, HA | ST789-IV [2B] | t547 | | CC7 | - | Egyir *et al*., 2015 |
|  |  |  |  | ST8-IV [2B]/V [5C2] | t024 | | CC8 | + |  |
|  |  |  |  | ST8-IV [2B] | t121 | | CC8 | + |  |
|  |  |  |  | ST8-V [5C2] | t024 | | CC8 | - |  |
|  |  |  |  | ST72-IV [2B]/V[5C2] | | t324, t537, t2649 | CC8 | - |  |
|  |  |  |  | ST239-III [3A] | t037 | | CC8 | - |  |
|  |  |  |  | ST247-I [1B] | t928 | | CC8 | - |  |
|  |  |  |  | ST36-II [2A] | t018 | | CC30 | - |  |
|  |  |  |  | ST508-V [5C2] | | t5132 | CC45 | - |  |
|  |  |  |  | ST88-IV [2B] | t186, t537 | | CC88 | - |  |
|  | 8 | Wound | HA | ST88-IV [2B] | ND | | CC88 | - | Wolter*s et* al., 2020 |

| **Country** | **No of MRSA** | **Sample** | **Setting** | **ST-SCC*mec*** | ***spa* type** | **MLST**  **Clonal complex** | **PVL** | **Reference** |
| --- | --- | --- | --- | --- | --- | --- | --- | --- |
| Kenya | 32 | Clinical | HA | ST5-II [2A] | t13150 | CC5 | ND | Omus*e et* al., 2016 |
|  |  |  |  | ST789-V [5C2] | t091 | CC7 | ND |  |
|  |  |  |  | ST8-IV [2B] | t104 | CC8 | ND |  |
|  |  |  |  | ST8-V [5C2] | t1476 | CC8 | ND |  |
|  |  |  |  | **ST241-III [3A]** | **t037**, t2029 | CC8 | ND |  |
|  |  |  |  | ST241-IV [2B] | t2029 | CC8 | ND |  |
|  |  |  |  | ST22-IV [2B] | t005, t022, t13149 | CC22 | ND |  |
|  |  |  |  | ST88-NT | t1339 | CC88 | ND |  |
|  |  |  |  | newST-NT | t3202 | - | ND |  |
|  |  |  |  | newST-1/IV/V | t345, t648, t689, t852 | - | ND |  |
|  |  |  |  | newST-IV [2B] | t293, t318, t9622 | - | ND |  |
|  | 8 | Clinical | HA | ST39-II [2A] | t007 | CC5 | - | Kyany'a *et al.,* 2019 |
|  |  |  |  | ST8-NT | t1476 | CC8 | - |  |
|  |  |  |  | **ST241-III [3A]** | **t037** | CC8 | - |  |
|  |  |  |  | ST4705-III [3A] | t2029 | CC8 | - |  |
|  |  |  |  | ST152-IVa [2B] | t355 | CC152 | - |  |
| Libya | 32 | Wound | HA | ST69-V [5C2] | t6065 | CC5 | - | Khemiri *et al.,* 2017 |
| Morocco | 17 | Nasal | CA | ST1-IV [2B] | t127 | CC1 | - | Mourabit *et al.,* 2017 |
|  |  |  |  | ST8-V [5C2] | t2658 | CC8 | - |  |
|  |  |  |  | **ST22-IV [2B]** | **t233**, t6397, t11293 | CC22 | - |  |
|  |  |  |  | ST398-IV [2B] | t011 | CC398 | - |  |
|  |  |  |  | ST (NT)-IV [2B] | t13247, t13248, t13249 | - | - |  |
| Nigeria | 8 | Nasal/cloacae | LA | ST5-V [5C2] | t002, t11469 | CC5 | - | Nworie *et al.,* 2017 |
|  |  |  |  | ST15-V [5C2] | t002, t084, t1164 | CC15 | - |  |
|  |  |  |  | ST121-V [5C2] | t002 | CC121 | - |  |
|  | 38 | Nasal | LA [26], CA [12] | ST88-IVa [2B] | t1603 | CC88 | - | Otalu *et al.,* 2018 |
|  | 30 | Chicken meat, nasal, environment | LA | **ST772-V [5C2]** | **t657** | CC1 | + | Ogundipe *et al.,* 2020 |
|  |  |  |  | ST789-V [5C2] | t091 | CC7 | - |  |
|  |  |  |  | ST8-Vc [5C2] | t2231, t12236 | CC8 | - |  |
|  |  |  |  | ST8-NT | t1476 | CC8 | - |  |
|  |  |  |  | ST121-IVa [2B] | t314 | CC121 | + |  |
|  |  |  |  | ST152-Vc [5C2] | t4690 | CC152 | + |  |

| **Country** | **No of MRSA** | **Sample** | **Setting** | **ST-SCC*mec*** | ***spa* type** | **MLST Clonal complex** | **PVL** | **Reference** |
| --- | --- | --- | --- | --- | --- | --- | --- | --- |
| São Tomé and Príncipe | 20 | Nasal | HA | ST1-V [5C2] | t590 | CC1 | + | Conceicao *et al.,* 2015a |
|  |  |  |  | ST8-V [5C2] | t451, t648 | CC8 | - |  |
|  |  |  |  | **ST88-IVa [2B]** | t186, **t786**, t1814 | CC88 | - |  |
|  | 29 | Nasal | HA | ST1-V [5C2] | t590 | CC1 | + | Conceicao *et al.,* 2015b |
|  |  |  |  | ST5-IVa [2B] | t105, t14047 | CC5 | - |  |
|  |  |  |  | ST105-II [2A] | t002 | CC5 | - |  |
|  |  |  |  | **ST8-IVg/V** | t064, **t451** | CC8 | - |  |
|  |  |  |  | ST88-IVa [2B] | t186, t786, t1814 | CC88 | - |  |
|  | 4 | Environmental surfaces | HA | ST8-VII [5C1] | t451 | CC8 | - | Aires-de-Sousa *et al.,* 2018 |
|  |  |  |  | **ST88-IVa [2B]** | t186, t786 | CC88 | - |  |
|  | 9 | Nasal | CA/HA | ST8-IVa [2B] | t121 | CC8 | + | Rodrigues *et al.,* 2018 |
|  |  |  |  | **ST8-V [5C2]** | **t008** | CC8 | - |  |
|  |  |  |  | ST88-IVa [2B] | t786 | CC88 | - |  |
| South Africa | 12 | Faecal, nasal, rinsate, carcass | LA | **ST612-IVd [2B]** | **t1257** | CC8 | + | Amoako *et al.,* 2019 |
|  |  |  |  | ST36-II [2A] | t018 | CC30 | + |  |
|  | 54 | Blood culture | HA | **ST5-NT** | **t045** | CC5 | ND | Abdulgader *et al.,* 2020 |
|  |  |  |  | ST239-III [3A] | t037 | CC8 | ND |  |
|  |  |  |  | ST612-IV [2B] | t1257 | CC8 | ND |  |
|  |  |  |  | ST22-IV [2B] | t032 | CC22 | ND |  |
|  |  |  |  | ST30-II [2A] | t012 | CC30 | ND |  |
|  | 17 | Clinical | HA | ST1-I [1B] | t465 | CC1 | * | Mahomed *et al.,* 2018 |
|  |  |  |  | ST1-IV [2B] | t465 | CC1 | * |  |
|  |  |  |  | ST8-I [1B] | t465 | CC8 | * |  |
|  |  |  |  | ST20-IV [2B] | t355, t16326 | CC20 | * |  |
|  |  |  |  | ST30-I [1B] | t030 | CC30 | * |  |
|  |  |  |  | ST30-IV [2B] | t030 | CC30 | * |  |
|  |  |  |  | ST45-I [1B] | t465 | CC45 | * |  |
|  |  |  |  | ST45-IV [2B] | t465 | CC45 | * |  |
|  |  |  |  | ST508-I [1B] | t603 | CC45 | * |  |
|  |  |  |  | **ST152-I [1B]** | t355, t715 | CC152 | * |  |
|  | 48 | Blood culture | CA/HA | ST5-III [3A] | t045 | CC5 | ND | Singh-Moodley *et al.,* 2020 |
|  |  |  |  | ST5-V [5C2] | t045 | CC5 | ND |  |
|  |  |  |  | ST5-NT | t045 | CC5 | ND |  |
|  |  |  |  | ST5-NT | t1257 | CC5 | ND |  |
|  |  |  |  | ST239-II [2A] | t1257 | CC8 | ND |  |
|  |  |  |  | ST239-III [3A] | t012 | CC8 | ND |  |
|  |  |  |  | ST239-III [3A] | t037 | CC8 | ND |  |
|  |  |  |  | ST239-III [3A] | t1257 | CC8 | ND |  |
|  |  |  |  | ST239-IV [2B] | t037 | CC8 | ND |  |
|  |  |  |  | ST239-NT | t037 | CC8 | ND |  |
|  |  |  |  | ST612-III [3A] | t012 | CC8 | ND |  |
|  |  |  |  | **ST612-IV [2B]** | **t064** | CC8 | ND |  |
|  |  |  |  | ST612-IV [2B] | t1257 | CC8 | ND |  |
|  |  |  |  | newST-IV [2B] | t1257 | CC8 | ND |  |
|  |  |  |  | ST22-IV [2B] | t012 | CC22 | ND |  |
|  |  |  |  | ST22-IV [2B] | t032 | CC22 | ND |  |
|  |  |  |  | ST4121-IV [2B] | t032 | CC22 | ND |  |
|  |  |  |  | ST36-II [2A] | t012 | CC30 | ND |  |
|  |  |  |  | ST36-II [2A] | t037 | CC30 | ND |  |
|  |  |  |  | ST36-II [2A] | t064 | CC30 | ND |  |
|  |  |  |  | ST36-III [3A] | t045 | CC30 | ND |  |
| Tunisia | 2 | Meat | LA | ST30-V [5C2] | t012 | CC30 | - | Chairat *et al.,* 2015 |
|  |  |  |  | ST398-IV [2B] | t4538 | CC398 | - |  |
| Uganda | 23 | Milk, milk products | LA | ST1-V [5C2] | t127 | CC1 | - | Asiimwe *et al.,* 2017b |
|  |  |  |  | ST97-V [5C2] | t3992 | CC97 | - |  |
|  |  |  |  | ST121-V [5C2] | t645 | CC121 | + |  |

LEGEND

*; Not clearly reported, -; absent +; present, ND; not detected

Predominant clone from each study is in bold
